# PP58 Central Precocious Puberty Clinical Practice Guidelines And Drug Use Profile In The Brazilian Unified Health System

**DOI:** 10.1017/S0266462325102778

**Published:** 2025-12-29

**Authors:** Rosângela Maria Gomes, Wallace Breno Barbosa, Marta da Cunha Lobo Souto Maior, Ana Carolina de Freitas Lopes, Luciene Fontes Schluckebier Bonan, Amanda Oliveira Lyrio

## Abstract

**Introduction:**

Central precocious puberty (CPP) is a rare condition, commonly characterized by early development of biochemical puberty and physical characteristics before the ages of eight (girls) and nine (boys) years. According to the Brazilian clinical practice guideline (CPG) the treatment of CPP includes gonadotropin-releasing hormone agonists. The objective of this study was to verify implementation of the CPP CPG using real-world drug dispensing data from the Brazilian Unified Health System.

**Methods:**

Data from drug dispensing in the Brazilian Unified Health System between 2013 and 2023 were extracted by a platform that creates follow-up cohorts for specific diseases (Open Room for Health Intelligence Situation). Only data for patients with an International Classification of Diseases, 10th Revision category of E22.8 were selected. All records that did not have encrypted user identification or that did not have an approved quantity of medication were excluded. A descriptive analysis was performed using PostgreSQL 24.2 to model the data.

**Results:**

A total of 147,045 individuals with CPP were treated with the recommended drugs in the Unified Health System over the study period. Most patients were female (94%). The mean age of patients at the beginning of treatment was 8.8 years (standard deviation 5.3). The most used drugs were leuprolide (80.2%), triptorelin (17.7%), goserelin (2.0%), and cyproterone acetate (0.2%). Leuprolide and triptorelin, with a biannual frequency of use, were included in the CPG at the end of 2022. Consequently, 1,905 patients used leuprolide (45 mg) in 2023 and 1,131 patients used triptorelin (22.5 mg).

**Conclusions:**

Data from the drug dispensing registry indicated that the CPP CPG is being implemented. Recently, this document was updated to recommend biannual frequency use of leuprolide and triptorelin to improve adherence to treatment. Data from the Open Room for Health Intelligence Situation platform can be an important source of information for evaluating the performance of these technologies.

